# Integrative taxonomy reveals first record of *Loxoscelesrufescens* (Dufour, 1820) (Araneae, Sicariidae) in the Philippines

**DOI:** 10.3897/BDJ.12.e117072

**Published:** 2024-02-19

**Authors:** Aimee Lynn A. Barrion-Dupo, Ireneo L. Lit, Jr., Camille Faith D. Duran, Ma. Francia Kyla M. Cammayo, Marnelli S. Alviola, Sheila Mae Q. Mercado, Cecille Ann L. Osio, Orlando L. Eusebio, Cristian C. Lucañas, Alberto T. Barrion

**Affiliations:** 1 Entomology Section, Museum of Natural History, University of the Philippines Los Baños (UPLB), Los Baños, Laguna, Philippines Entomology Section, Museum of Natural History, University of the Philippines Los Baños (UPLB) Los Baños, Laguna Philippines; 2 Institute of Biological Sciences, College of Arts and Sciences, University of the Philippines Los Baños (UPLB), Los Baños, Laguna, Philippines Institute of Biological Sciences, College of Arts and Sciences, University of the Philippines Los Baños (UPLB) Los Baños, Laguna Philippines; 3 Department of Biology, College of Science, De La Salle University, Taft, Manila, Philippines Department of Biology, College of Science, De La Salle University Taft, Manila Philippines

**Keywords:** caves, *Loxoscelesrufescens*, loxoscelism, Mediterranean recluse

## Abstract

**Background:**

The spider family Sicariidae Keyserling, 1880 represented by the synanthropic Mediterranean recluse spider, *Loxoscelesrufescens* (Dufour, 1820), is reported in the Philippines for the first time, based on morphological and molecular data. The introduced spider was observed in a small cave (Kamantigue Cave) in Lobo, Batangas Province. Considering the medical importance of this spider, the proximity of its habitat to human habitation and tourist sites poses a potential public health concern.

**New information:**

This study reports on the first record of the family Sicariidae in the Philippines and the fourth recorded occurrence of *L.rufescens* in Southeast Asia.

## Introduction

Spiders are amongst the most widely distributed organisms in the world, with currently more than 50,000 recorded valid species belonging to 132 families (World Spider Catalog 2023). In the Philippines, the documented spider fauna is usually limited to agricultural areas (Barrion and Litsinger 1995). Studies of species in cave and karst environments are relatively recent, the most notable being those of Barrion-Dupo et al. (2014), Rasalan et al. (2015), Maandig et al. (2017) and Rasalan and Barrion-Dupo (2019). Fieldwork and other research activities in several areas within southern Tagalog (CALABARZON) Region in Luzon are steadily accumulating material towards more comprehensive treatment of spiders and allies in the area. Here, we report the first field observations of a notable population of *Loxoscelesrufescens* Dufour, 1820 (Dufour 1820) found in a cave in Lobo, Batangas, Philippines.

*Loxosceles* Heineken & Lowe, in Lowe (1832), also known as recluse or violin spiders, due to the distinctive violin-shaped mark on the cephalothorax, are characterised by their slanted legs when at rest and by having six eyes arranged in pairs (Vetter and Visscher 1998, Lotz 2017, Trivedi and Dal 2019). The genus currently consists of 143 species (World Spider Catalog 2023). These spiders are known to inflict dangerous bites, known as loxoscelism which can cause necrotic skin lesions, dermonecrosis and skin ulceration (Nentwig et al. 2017, Zamani et al. 2020). Alleged deaths caused by *L.rufescens* were reported in Europe and in Thailand (Pezzi et al. 2016, Nentwig et al. 2017). However, both of the claims did not confirm that the bite was from *L.rufescens*.

*Loxoscelesrufescens* or the Mediterranean recluse probably originated from North Africa and became naturalised in the circum-Mediterranean Region (Gertsch and Ennik 1983, Duncan et al. 2010), but is now considered cosmopolitan as it has already spread across various temperate and tropical countries and regions like the USA, Asia, Australia, Atlantic Region, Madagascar, Hawaiian Islands and Socotra Islands in Yemen (Gertsch and Ennik 1983, Vetter 2008, Planas et al. 2014, Hula and Niedobová 2020). In Southeast Asia, it was recently reported to occur in caves and environs in Thailand, Laos and Malaysia (Jäger 2007, Duncan et al. 2010, Chomphuphuang et al. 2016).

## Materials and methods

Specimens were collected from Kamantigue Cave, Barangay Biga, Lobo, Batangas on 10-13 September 2022 and 9-13 November 2022 under the Department of Environment and Natural Resources Wildlife Gratuitous Permit (DENR WGP): R4A-WGP-2021-BAT-006. Photographs of spiders in their natural habitat were taken using a Nikon D3100 with a macro lens. All specimens were deposited in the Entomology Section, Museum of Natural History, University of the Philippines Los Baños (UPLB-MNH).

**Figure 1.**
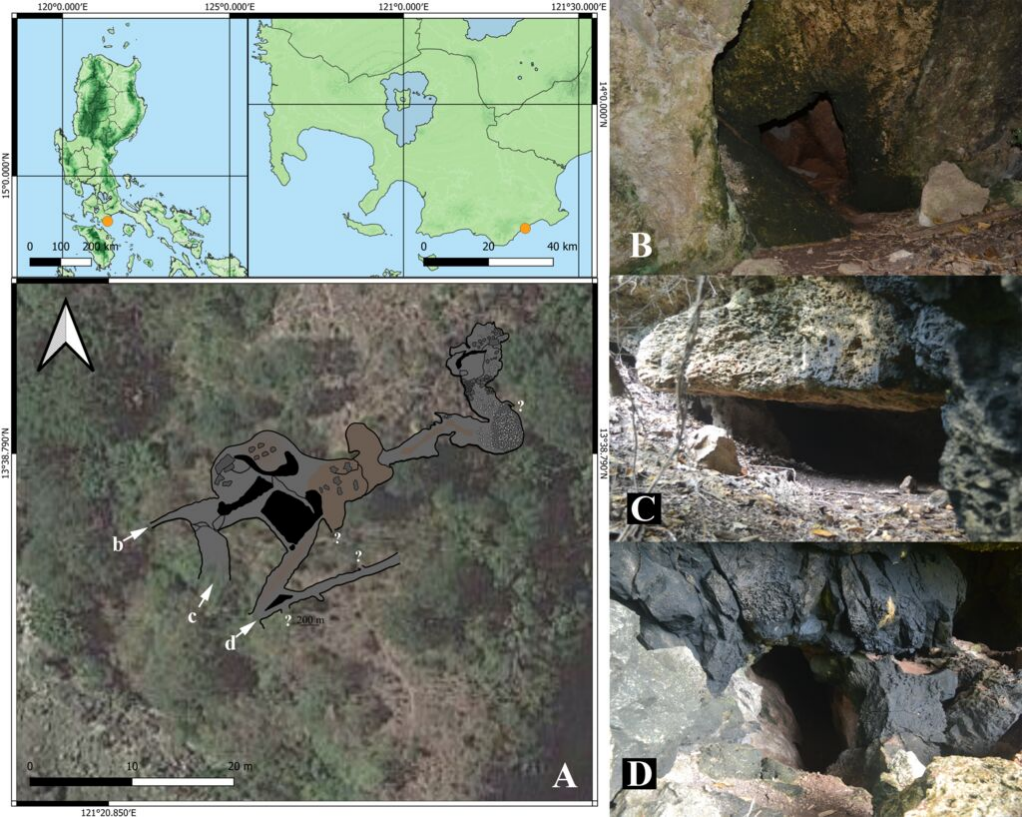
Map of Kamantigue Cave in Lobo, Batangas, Philippines (**A**) and cave entrances (**B-D**). Cave map by DENR CENRO-Lipa, Batangas; b, c, d = entrance; ? - unexplored areas.

Kamantigue Cave (Fig. 1) is located a few metres from the seashore and a few metres adjacent to residential houses. Based on DENR Memorandum Circular No. 2021-193, the cave is classified as Class I due to its extremely hazardous conditions.

The cave has three low and narrow entrances and multiple narrow chambers with abundant guano deposits. The temperature inside the cave is notably high and air flow is very limited. Portions of the cave have collapsed and eroded rocks blocked some of its chambers.

Collected specimens were submerged in 80% ethanol, examined and measured under a Nikon SMZ 800 stereomicroscope, fitted with an ocular micrometer. Body measurements followed the standard procedure adopted by Barrion and Litsinger (1995). All measurements are in mm. Male and female copulatory organs were examined prior to dissection. The right pedipalp of the male was cut and photographed apically, prolaterally and retrolaterally using an Olympus Z61 stereomicroscope mounted with a digital camera. The female epigynum was cut from the abdomen and cleared in 10% potassium hydroxide (KOH) for 15-20 mins. It was then washed with distilled water three times and transferred to glycerine.

Confirmation of species identification was also performed via molecular analysis of the mitochondrial cytochrome oxidase subunit I (*COI*) gene. Genomic DNA was extracted from the whole specimen using Wizard® Genomic DNA Purification Kit (Promega, Madison, WI, USA), following the manufacturer’s protocol. The *COI* gene was amplified using the forward (5′-GGAGGATTTGGAAATTGATTAGTTCC-3′) and reverse (5′-CCCGGTAAAATTAAAATATAAACTTC-3′) primers designed by Simon et al. (1994). The 25-µl PCR reaction tubes, each containing the 1× GoTaq® Colorless Master Mix (Promega), 0.1 mM forward and reverse primer, ≤ 250 ng DNA template and nuclease-free water, were placed in the Bioer GeneExplorer™ Thermal Cycler (Alpha Laboratories, Eastleigh, UK). The cycling profile was as follows: initial denaturation at 95°C for 2 min; 35 cycles of denaturation at 95°C for 30 s, annealing at 95°C for 30 s and extension at 46°C for 1 min; final extension at 72°C for 2 min; and holding at 4°C for 10 min. The PCR products were sent to Macrogen (Seoul, South Korea) for standard DNA sequencing.

The raw sequences were preprocessed by trimming the ends to remove the low-quality bases (< 10 quality scores) and editing the ambiguous bases using Chromas 2.6.6 (http://technelysium.com.au/wp/chromas/). The nucleotide basic local alignment search tool (BLASTn, Altschul et al. (1990)) was used to determine sequence similarity with the publicly available sequences in the National Center for Biotechnology Information (NCBI) database (Table 1). Sequence alignment was performed using MUSCLE (Edgar 2004), followed by phylogenetic analysis using MEGA 11 software (Tamura et al. 2021). A consensus tree was generated using the unweighted pair group method with arithmetic mean (UPGMA) and 1,000 bootstrap replications.

## Taxon treatments

### Loxosceles rufescens

(Dufour, 1820)

546FABA6-6D99-5D3D-9F54-EF113A2A68BF

*Scytodesrufescens* Dufour, 1820 - Dufour (1820): 203; For full synonymy, see World Spider Catalogue.

#### Materials

- **Type status:** Other material. **Occurrence:** catalogNumber: ARA-00526, 527; 528-530; individualCount: 5; sex: 2 males, 3 females; lifeStage: adult; preparations: in EtOH; occurrenceID: 617D84B3-4CE3-58F4-980F-0AD6ABA92187; **Location:** country: Philippines; stateProvince: Batangas; municipality: Lobo; locality: Brgy. Biga, Kamantigue Cave; verbatimLocality: PHILIPPINES: Luzon, Batangas, Lobo, Biga: Kamantigue Cave; **Identification:** identificationID: Loxoscelesrufescens; identifiedBy: NICER P3; **Event:** eventDate: 11-09-2022; year: 2022; month: 09; day: 11; habitat: Kamantigue Cave; **Record Level:** type: PhysicalObject; institutionID: UPLB MNH; institutionCode: UPLBMNH
- **Type status:** Other material. **Occurrence:** catalogNumber: ARA-00531, 532, 533, 534; individualCount: 4; sex: 1 male, 2 females, 1 juvenile; lifeStage: 3 adults, 1 juvenile; preparations: in EtOH; occurrenceID: 31CBAEC6-CBA0-5A85-B4C4-BC53671C4089; **Location:** country: Philippines; stateProvince: Batangas; municipality: Lobo; locality: Brgy. Biga, Kamantigue Cave; verbatimLocality: PHILIPPINES: Luzon, Batangas, Lobo, Biga: Kamantigue Cave; **Identification:** identificationID: Loxoscelesrufescens; identifiedBy: NICER P3; **Event:** eventDate: 19-11-2022; year: 2022; month: 11; day: 19; habitat: Kamantigue Cave; **Record Level:** type: PhysicalObject; institutionID: UPLB MNH; institutionCode: UPLBMNH

#### Description

**Figure 2.**
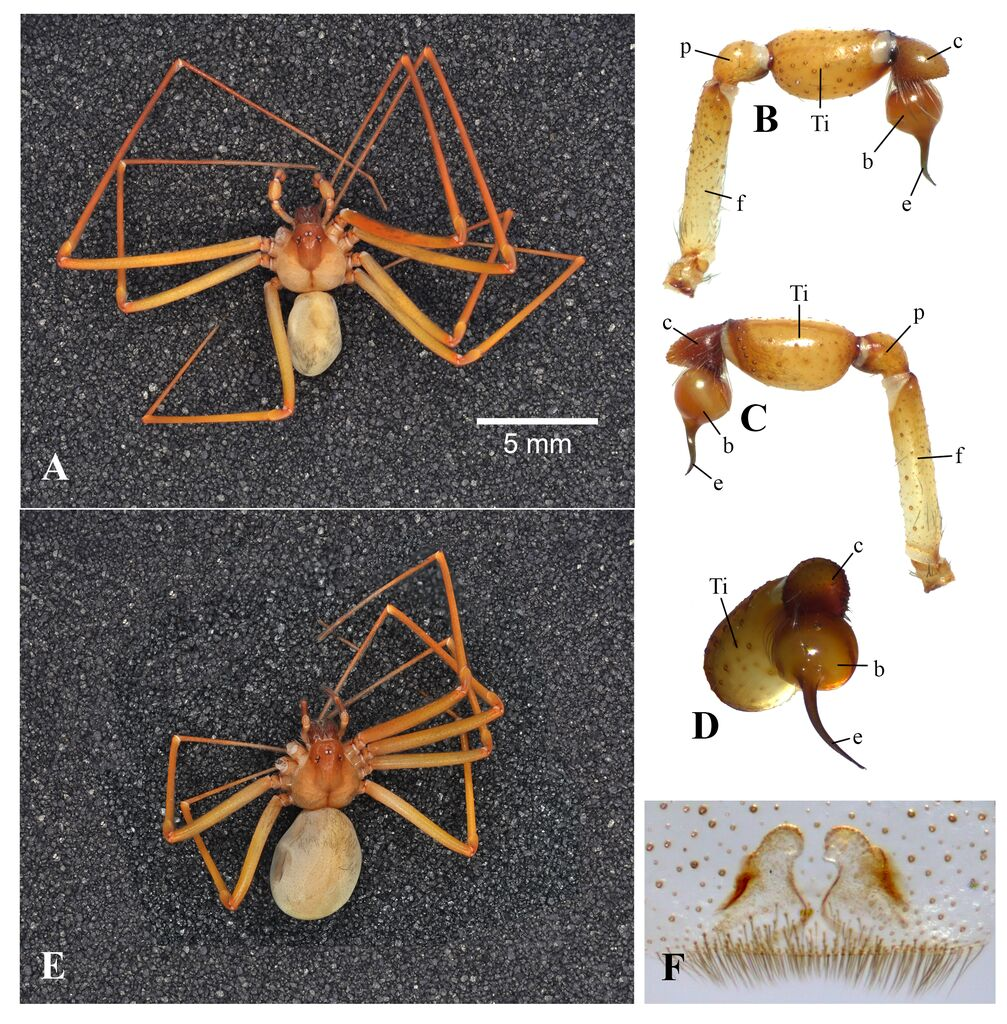
*Loxoscelesrufescens* (Dufour, 1820), male (**A-D**) and female (**E-F**); Habitus, dorsal view (**A, E**); palp: prolateral (**B**), retrolateral (**C**), apical (**D**); spermatheca (**F**). Abbreviations: f - femur, p - patella, Ti - tibia, c - cymbium, b - bulb, e - embolus.

Male (Fig. 2A). Total length 8.05 + 0.92. Carapace: length 4.30 + 0.14, width 3.40 + 0.57, height 1.75 + 0.21. Abdomen: length 3.75 + 1.06, width 2.18 + 0.46, height 2.08 + 0.18. Pedipalp length 3.75; femur 1.40 long; patella 0.45; tibia 1.00 long, 0.60 wide; cymbium 0.50 long, 0.40 wide; bulb 0.40 long. 0.45 wide and embolus 0.50 long.; palpal tibia length/width ratio 1.67. Embolus (1.19 + 0.01) as long as width of globular bulb (1.16 + 0.01).

Carapace pale orange-brown marked with dorsal dark orange-brown violin-shaped marking. Eyes six in three dyads in a recurved transverse row. Sternum pale yellowish to cream. Chelicerae, labium and maxillae reddish-brown. Legs orange-brown. Abdomen ground colour cream brown to greyish-brown, with short, grey setae. Leg formula 2-1-4-3 (Table 2). Male palp. Cymbium noticeably shorter than tibia length (0.40:1.00), slightly longer than palp bulb. Embolus possessing a thin cylindrical shaft towards apex (Figs. 2B-2D). Female. Habitus as in (Fig. 2E) Total length 8.10 + 1.63. Carapace: length 3.38 + 0.62, width 2.83 + 0.42, height 1.86 + 0.15. Abdomen: length 4.73 + 1.17, width 2.85 + 0.66, height 3.05 + 0.90. Colouration and eye arrangement same as male. Leg formula as in male (Table 2). Spermatheca (Fig. 2F) short and rounded distally, its anterior end rounded and directed towards each other (converging) and basal area relatively wide.

#### Distribution

Southern Europe, northern Africa to Afghanistan, Iran. Introduced to the USA, Mexico, Peru, Macronesia, South Africa, India, Yemen, China, Japan, Korea, Laos, Malaysia, Thailand, Australia, Hawaii and Philippines (new record).

#### Notes

This represents a new record of the family Sicariidae Keyserling, 1880 and species in the Philippines. It can be distinguished from other spider families in the Philippines by the six eyes arranged in three dyads in a recurved row, relatively flat carapace, rounded abdomen and tarsal claws two (compared to Scytodidae: humped carapace, tarsal claws 2-3; and 6-eyed Pholcidae: eyes arranged in two distinct triads, abdomen usually elongate and narrow, tarsal claws 2-3).The Philippine specimens exhibit the typical spermatheca and male palp features of the *rufescens*-species group (Binford et al. 2008, Fukushima et al. 2017). Brignoli (1969) and Zamani et al. (2020) noted several variations on the epigyne of *L.rufescens* in Mediterranean, Iran, Afghanistan and Turkmenistan species, but refrained from describing them as distinct species without additional specimens.

The spermatheca of Philippine specimens are short and rounded distally with reduced or absent spermathecal bilobation, similar to those from the Balkan Peninsula (Naumova and Deltshev 2021), Mexico (Valdez-Mondragón et al. 2018) and from Hormozgan in Iran (Zamani et al. 2020), but slightly differs in the size of the inner receptacle lobe. It also closely resembles the Australian specimen recovered in the Iberian Clade by Duncan et al. (2010).

Similarly, the male palp of Philippine specimens conforms with the report of Lotz (2017) of *L.rufescens*: (a) the short cymbium; (b) cymbium slightly longer than palp bulb; (c) ratio of palp tibia length/height is 1.67; (d) palp cymbium noticeably shorter than the tibia [0.40:1.00]; and (d) embolus possessing a thin cylindrical shaft towards the apex (Fig. 2B-D).

Overall, the examined morphological characters of the Philippine species conform with the present description of *L.rufescens* as presented in Lotz (2017), Valdez-Mondragón et al. (2018),Zamani et al. (2020), and Naumova and Deltshev (2021).

Furthermore, the results of the molecular analysis corroborate those obtained using classical morphological techniques. The BLASTn results of the *COI* sequences generated from four Philippine spider specimens (412–433 bp long) reveals significantly high similarity (percent identity = 98–100%) with those of *L.rufescens*. Meanwhile, the pairwise distance between the Philippine samples and those from Mediterranean samples ranges from zero to nearly 0.1 (Table 3). Although the taxonomy of *L.rufescens* is not yet fully resolved (Duncan et al. 2010), the Philippine specimens closely match those presently considered as *L.rufescens*.

Interestingly, distances between Philippine specimens and those from India (Maharashtra), Portugal (Porto Santo) and Spain (Sagunt) were recovered to be zero. Historically around the 18^th^ century, there is an existing trade route between India - Philippines (Seshan 2006, Eang 2011), countries which were occupied by Portugal and Spain, respectively. Given this, it is possible that the Philippine populations have been introduced via this trade route. This hypothesis can be tested if other specimens of *L.rufescens* could be observed in areas following this trade route; unfortunately, molecular information from specimens in Perak, Malaysia is lacking. However, the distance between the eyes of females from Philippines resemble those from Malaysia (eye dyads separated by ~ 2-2.5 median eye diameter), but with slightly more recurved eyes (Duncan et al. 2010).

Additionaly, distances between Philippine specimens and those from Australia (Adelaide) and USA (New York) were also recovered to be zero. Duncan et al. (2010) recovered both specimens to belong to the Iberian clade of *L.rufescens*. However, this may suggest an alternative route of introduction for the Philippine population.

On the other hand, Philippine specimens were recovered to be distant from those from Guangxi, China suggesting that they may have followed different routes of introduction. Luo and Li (2015) suggested that *L.rufescens* was introduced to China around 42,710-46,008 years ago which coincides with the movement of modern humans to East and South-East Asia.

**Figure 3.**
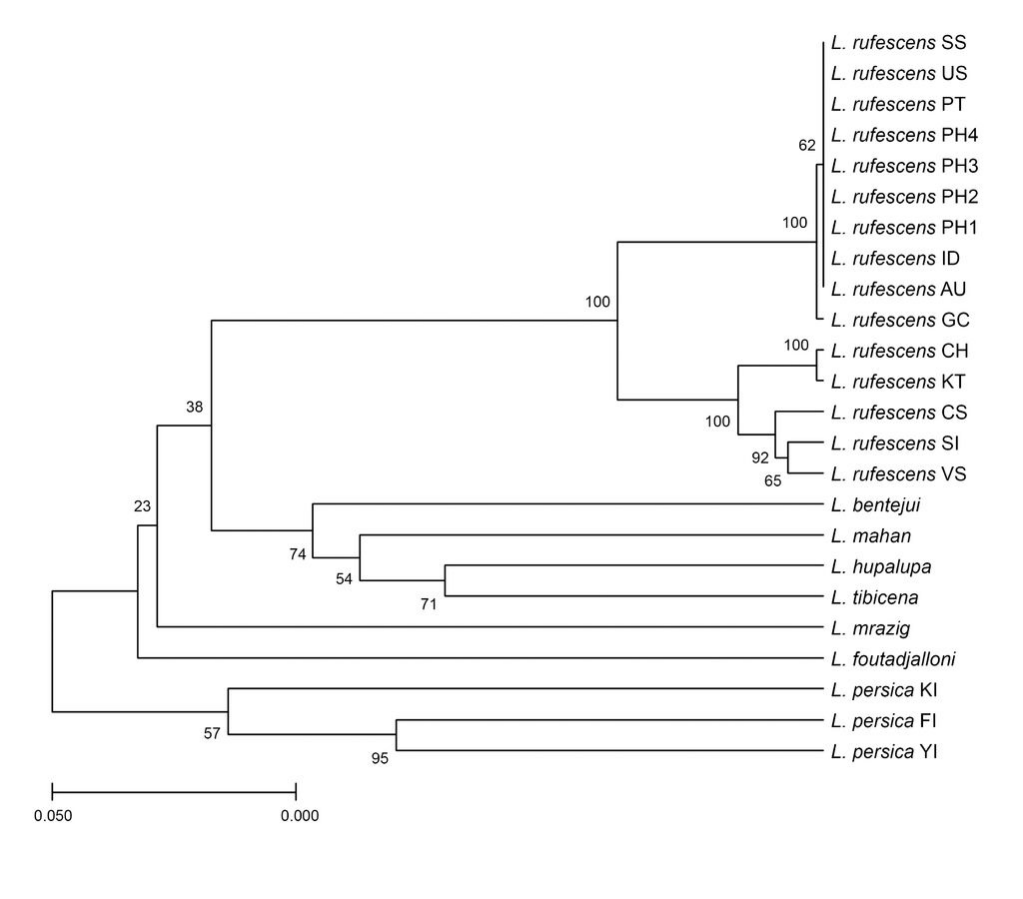
UPGMA tree generated using the *COI* sequences of the four Philippine *Loxoscelesrufescens* (Dufour, 1892) specimens and 20 other *Loxosceles* species. Evolutionary distances were calculated using the Tamura-Nei parameter model with 1,000 bootstrap iterations. AU – Adelaide, Australia; CH – Guangxi, China; CS – Cabo de Gata, Spain; FI – Fars, Iran; GC – Subida San Felipe, Gran Canaria; ID - Maharashra, India; KT – Kepez, Turkey; PH – Batangas, Philippines; PT – Porto Santo, Portugal; SI – Sardinia, Italy; SS – Sagunt, Spain; US – New York, USA; VS – Vilamarxant, Spain; YI – Yazd, Iran.

Phylogenetic analysis shows three major clusters consisting of *L.rufescens* specimens (100% bootstrap support), Canarian species (74% bootstrap) and *L.persica* (57% bootstrap) (Fig. 3). In the *L.rufescens* cluster, the specimens from Kamantigue Cave, Lobo, Batangas (PH1–PH4) are grouped together with specimens from Australia (AU), Gran Canaria (GC), India (ID), Portugal (PT), Spain (SS) and America (US). This group is sister to a clade of *L.rufescens* specimens from China (GH), Turkey (KT), Italy (SI) and Spain (CS, VS).

## Discussion

The genus *Loxosceles* Heineken & Lowe, in Lowe (1832), is known to occur in the temperate areas of South Africa, in the Tropics, in the Mediterranean Region and southern Europe. In America, it ranges from the temperate and tropical regions of North and South America (Gertsch and Ennik 1983). *Loxoscelesrufescens* originated from North Africa, probably Morocco and transported within the Mediterranean Basin through human agencies and its own means (Duncan et al. 2010, Planas et al. 2014, Nentwig et al. 2017). The unintentional, human-mediated introduction of this spider also brought them to the United States (Gertsch and Ennik 1983, Massa et al. 2018). Currently, the species is now spread through the islands of the Atlantic, Madagascar, Hawaii and in the areas of Australia, Mexico, Iran, Afghanistan, Peru, Macaronesia and South Africa (Chomphuphuang et al. 2016, Gertsch and Ennik 1983, World Spider Catalog 2023Chomphuphuang et al. 2016,). In Asia, it has been reported to occur in Iran (Zamani et al. 2020), India (Trivedi and Dal 2019, Sahoo et al. 2022), China (Chen and Gao 1990), Russia (Dunin 1992), Taiwan (Song et al. 1999), Japan (Ono 2009) and South Korea (Namkung 2003). In Southeast Asia, it is reported to be observed in caves in Laos (Jäger 2007) and Thailand (Chomphuphuang et al. 2016) and karst environs in Malaysia (Duncan et al. 2010). Zamani et al. (2020) noted the possibility that some of those populations (i.e. those from China) might not be conspecific. Nonetheless, with this distribution, the species is now considered cosmopolitan.

*Loxoscelesrufescens* are usually found in caves, under rocks and leaf litter in its Mediterranean distribution (Simon 1914). Reports outside its region of origin, found them living in favour of urban areas, such as residential houses, apartments, university buildings, basements, tunnels and other cave-like human-made structures. Their microhabitats include corner walls, storage boxes, electric meter boxes and crevices of unused cupboards (Gertsch and Ennik 1983, Trivedi and Dal 2019). They are also found under logs, leaves of wild shrubs, leaf litter and rocks near urban environments (Sahoo et al. 2022).

In more recent studies outside the Mediterranean Region, *L.rufescens* was also found in caves. In Laos, they were documented to occur in two caves, The Pak Ou (Tham Phun) and Tham Sing Mang Caves, which are both dry caves (Jäger 2007). The species was also found in the Tum-Wangpra Cave in Thailand (Chomphuphuang et al. 2016). Interestingly, these caves are all reported to be dry and have either high temperature or have warmer ground surface.

**Figure 4.**
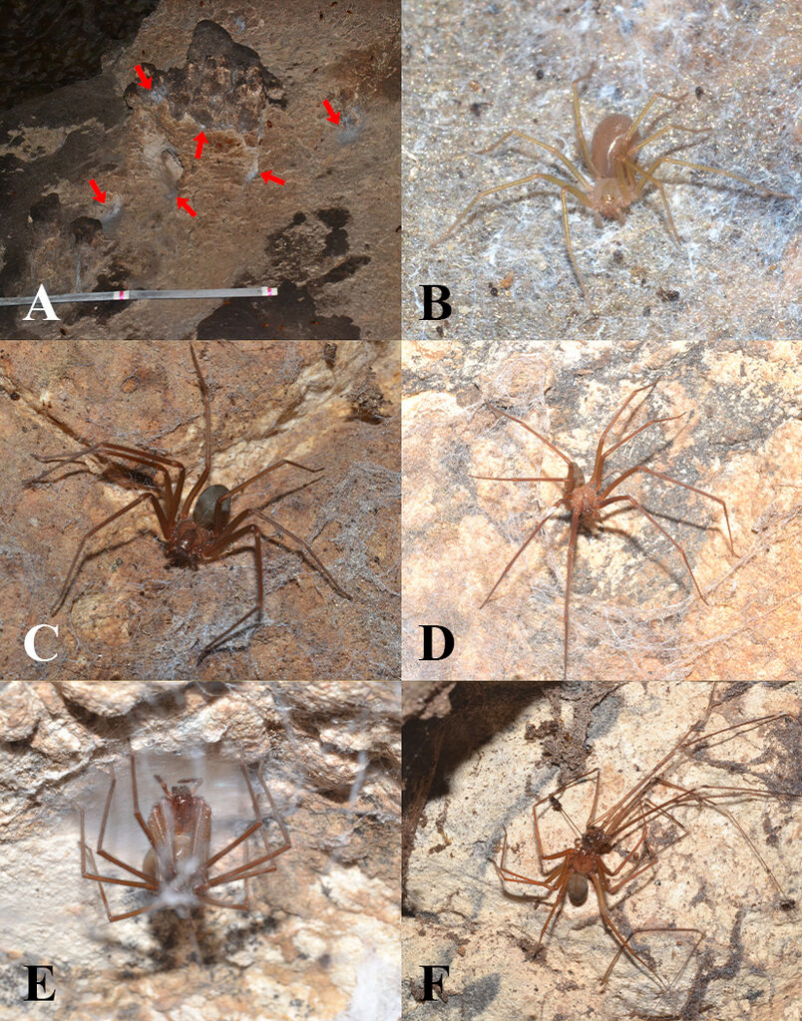
*Loxoscelesrufescens* (Dufour, 1820) in Kamantigue Cave. **A** web in cave wall; **B** Juvenile; **C** Male; **D** Female; **E** Female with eggs; **F** Female feeding on a pholcid, *Smeringopuspallidus* (Blackwall, 1858).

The abiotic conditions (e.g. precipitation and temperature) used by Taucare‐Ríos et al. (2018) to create the global model of distribution for *L.rufescens* predicted that a probable sink population may be present in north Luzon, particularly, the Ilocos Sur area. Our current observation deviates from this model, as *L.rufescens* was collected in southern Luzon, specifically in the Kamantigue Cave of Barangay Biga, Lobo, Batangas (Fig. 4). The presence of this spider beyond its native range may be an unintentional introduction as chance passengers during travel, trade and transport.

The spider can be easily spread through human means as they are able to resist long periods of starvation and they are highly synanthropic (i.e. live in close association with humans and benefits from their habitats, surroundings and activities; Massa et al. (2018)). They prefer to hide in wooden objects, cardboard boxes, containers and other structures that have small crevices, which make them easy to transport along with other goods (Nentwig et al. 2017).

**Potential invasiveness in the environment**. The cave where *L.rufescens* has been found is inhabited by another invasive species, the American cockroach, *Periplanetaamericana* (L.), which are known prey for these spiders (Greene et al. 2009). However, individuals were observed to feed also on the pholcid spider *Smeringopuspallidus* (Blackwall 1858) (Fig. 4F) and even other individuals of *L.rufescens* and, hence, may be cannibalistic. Despite being dominated by other invasive species, several native and potentially endemic species are possibly preyed upon by *L.rufescens*. Unidentified Scytodidae and guano moths are observed in guano-rich chambers, while some unidentified Gnaphosidae and a cave-dwelling cockroach, *Nocticola* sp. were observed on guano-poor chambers. Interestingly, the cave-associated whip spider, *Charon* sp., was observed to be relatively abundant in rock crevices outside, but nearly absent inside the cave. The spider is also known to be potentially harmful to humans (Vetter 2008, Nentwig et al. 2017).

Despite their invasiveness and potential harm, *L.rufescens* do not disperse on their own means easily. All *Loxosceles* species have low dispersal capacity and do not balloon like other spiders (Vetter 2008, Greene et al. 2009, Massa et al. 2018). They are usually sedentary and prefer to stay in the same location for long periods of time (Massa et al. 2018). In casual conversations with the locals from the residential houses near the cave, they said that there are no sightings of *L.rufescens* in their houses.

**Medical importance**. The genus *Loxosceles* is amongst the known dangerous spiders in the world (Sadeghi et al. 2017). All *Loxosceles* species are considered medically important due to their ability to cause skin injury. Their bites can cause local erythema and necrotic skin reactions. In rare cases, it can lead to “loxoscelism”, a disease that causes myalgia, arthralgia, haemolysis, haemoglobinuria, acute renal failure and amputation and, very rarely, death (Vetter 2008, Bajin et al. 2011, Köse et al. 2021).

In most cases involving *Loxoscelesrufescenes*, most bites do not result in serious skin injuries (Nentwig et al. 2017). Most are typically mild and self-healing (Vetter 2008, Nentwig et al. 2017). Bites are described as painless and usually occur at night due to the nocturnal nature of *Loxosceles* spiders. Typically, these spiders only bite for defensive purposes.

**Implication in cave classification and management.** Kamantigue Cave located at Barangay Biga, Lobo, Batangas is currently classified as Class I due to extremely hazardous conditions, such as its bad air condition and presence of rock fall (Department of Environment and Natural Resources 2021). The presence of a viable population of medically important *L.rufescens* is an additional reason to restrict caving activities considering that therapy for Mediterranean recluse spider envenomation still needs development (Sadeghi et al. 2017).

## Supplementary Material

XML Treatment for Loxosceles rufescens

## Figures and Tables

**Table 1.** Localities of *Loxosceles* spp. sequences used for phylogenetic analysis.

**Species**	**Locality**	**Accession**	**Reference**
*L.bentejui*	Agaete, Gran Canaria	KF669917	Planas and Ribera (2014)
*L.foutadjalloni*	Segueya, Guinea	GQ279239	Duncan et al. (2010)
*L.hupalupa*	Igualero, La Gomera	KF670003	Planas and Ribera (2014)
*L.mahan*	Teguitar, Fuerteventura	KF669928	Planas and Ribera (2014)
*L.mrazig*	Douz, Tunisia	FJ986179	Ribera and Planas (2009)
*L.persica* FI	Kaviri Cave, Ghaemiye, Fars, Iran	MF467576	Tahami et al. (2017)
*L.persica* KI	Ker Palang Cave, Mal Agha, Khuzestan, Iran	MF467577	Tahami et al. (2017)
*L.persica* YI	Khaneh Khoda Cave, Harat, Yazd, Iran	MF467575	Tahami et al. (2017)
*L.rufescens* AU	Adelaide, Australia	GQ279229	Duncan et al. (2010)
*L.rufescens* CH	Guangxi, China	MH382645	Li and Li (2018)
*L.rufescens* CS	Cabo de Gata, Spain	KF717002	Planas and Ribera (2014)
*L.rufescens* GC	Subida San Felipe, Gran Canaria	KF717009	Planas and Ribera (2014)
*L.rufescens* ID	Maharashtra, India	KT383752	Gaikwad et al. unpublished data
*L.rufescens* KT	Kepez, Turkey	KJ560582	Planas et al. (2014)
*L.rufescens* PH1	Kamantigue Cave, Lobo, Batangas, Philippines	OR835851	this study
*L.rufescens* PH2	Kamantigue Cave, Lobo, Batangas, Philippines	OR835852	this study
*L.rufescens* PH3	Kamantigue Cave, Lobo, Batangas, Philippines	OR835853	this study
*L.rufescens* PH4	Kamantigue Cave, Lobo, Batangas, Philippines	OR835854	this study
*L.rufescens* PT	Porto Santo, Portugal	KF717006	Planas and Ribera (2014)
*L.rufescens* SI	Sardinia, Italy	KJ560589	Planas et al. (2014)
*L.rufescens* SS	Sagunt, Spain	KF717003	Planas and Ribera (2014)
*L.rufescens* US	New York, USA	GQ279227	Duncan et al. (2010)
*L.rufescens* VS	Vilamarxant, Spain	KF717004	Planas and Ribera (2014)
*L.tibicena*	Cumbre Arico, Tenerife	KF669925	Planas and Ribera (2014)

**Table 2.** Leg measurements of *Loxoscelesrufescens* (Dufour, 1820) from the Philippines.

Sex	Leg	Femur	Patella	Tibia	Metatarsus	Tarsus	Total
Male	1	7.25	0.88	8.30	8.65	1.33	26.40
	2	8.95	1.08	9.20	9.25	1.65	30.13
	3	6.55	0.90	7.55	7.45	1.45	23.90
	4	7.10	0.85	7.00	7.50	1.68	24.13
	Pedipalp	1.33	0.28	0.75	-	1.33	3.68
Female	1	5.33	0.91	5.80	5.00	1.09	18.13
	2	5.85	0.96	6.28	5.58	1.18	19.84
	3	4.85	1.00	4.45	4.83	1.05	16.21
	4	5.31	0.95	5.43	6.18	1.13	18.99
	Pedipalp	1.21	0.35	0.83	-	1.21	3.60

**Table 3.** Pairwise Distance Matrix of *COI* sequences of *Loxoscelesrufescens* (Dufour, 1820) from the Philippines and other localities: AU – Adelaide, Australia; CS – Cabo de Gata, Spain; CH – Guangxi, China; GC – Subida San Felipe, Gran Canaria; ID – Maharastra, India; KT – Kepez, Turkey; PH – Batangas, Philippines; PT – Porto Santo, Portugal; SI – Sardinia, Italy; SS – Sagunt, Spain; US – New York, USA; VS – Vilamarxant, Spain.

	**AU**	**CH**	**CS**	**GC**	**ID**	**KT**	**PH1**	**PH2**	**PH3**	**PH4**	**PT**	**SI**	**SS**	**US**	**VS**
**AU**		0.0221	0.0248	0.0024	0.0000	0.0229	0.0000	0.0000	0.0000	0.0000	0.0000	0.0224	0.0000	0.0000	0.0201
**CH**	0.0731		0.0105	0.0241	0.0249	0.0029	0.0237	0.0249	0.0242	0.0238	0.0237	0.0113	0.0237	0.0225	0.0111
**CS**	0.0851	0.0314		0.0259	0.0276	0.0111	0.0254	0.0267	0.0259	0.0255	0.0254	0.0077	0.0254	0.0252	0.0076
**GC**	0.0025	0.0832	0.0916		0.0028	0.0249	0.0022	0.0023	0.0023	0.0022	0.0022	0.0244	0.0022	0.0025	0.0223
**ID**	0.0000	0.0806	0.0944	0.0286		0.0248	0.0000	0.0000	0.0000	0.0000	0.0000	0.0259	0.0000	0.0000	0.0241
**KT**	0.0763	0.0023	0.0340	0.0816	0.0806		0.0247	0.0257	0.0250	0.0245	0.0244	0.0113	0.0244	0.0232	0.0118
**PH1**	0.0000	0.0805	0.0889	0.0023	0.0000	0.0834		0.0000	0.0000	0.0000	0.0000	0.0239	0.0000	0.0000	0.0218
**PH2**	0.0000	0.0846	0.0934	0.0024	0.0000	0.0877	0.0000		0.0000	0.0000	0.0000	0.0251	0.0000	0.0000	0.0230
**PH3**	0.0000	0.0822	0.0908	0.0024	0.0000	0.0852	0.0000	0.0000		0.0000	0.0000	0.0244	0.0000	0.0000	0.0219
**PH4**	0.0000	0.0806	0.0889	0.0023	0.0000	0.0835	0.0000	0.0000	0.0000		0.0000	0.0239	0.0000	0.0000	0.0219
**PT**	0.0000	0.0805	0.0889	0.0023	0.0000	0.0835	0.0000	0.0000	0.0000	0.0000		0.0239	0.0000	0.0000	0.0218
**SI**	0.0765	0.0340	0.0191	0.0865	0.0877	0.0339	0.0837	0.0880	0.0855	0.0840	0.0838		0.0239	0.0227	0.0062
**SS**	0.0000	0.0805	0.0889	0.0023	0.0000	0.0835	0.0000	0.0000	0.0000	0.0000	0.0000	0.0838		0.0000	0.0219
**US**	0.0000	0.0741	0.0862	0.0025	0.0000	0.0773	0.0000	0.0000	0.0000	0.0000	0.0000	0.0776	0.0000		0.0204
**VS**	0.0673	0.0338	0.0190	0.0778	0.0806	0.0364	0.0751	0.0789	0.0766	0.0753	0.0751	0.0141	0.0775	0.0682	
